# On the Role of Mechanics in Chronic Lung Disease

**DOI:** 10.3390/ma6125639

**Published:** 2013-12-04

**Authors:** Mona Eskandari, Martin R. Pfaller, Ellen Kuhl

**Affiliations:** Department of Mechanical Engineering, Stanford University, 496 Lomita Mall, Stanford, CA 94305, USA; E-Mails: eskandar@stanford.edu (M.E.); pfaller@stanford.edu (M.R.P.)

**Keywords:** material modeling, biomaterials, instability, buckling, folding, growth, remodeling, finite element method, airway wall remodeling, chronic lung disease

## Abstract

Progressive airflow obstruction is a classical hallmark of chronic lung disease, affecting more than one fourth of the adult population. As the disease progresses, the inner layer of the airway wall grows, folds inwards, and narrows the lumen. The critical failure conditions for airway folding have been studied intensely for idealized circular cross-sections. However, the role of airway branching during this process is unknown. Here, we show that the geometry of the bronchial tree plays a crucial role in chronic airway obstruction and that critical failure conditions vary significantly along a branching airway segment. We perform systematic parametric studies for varying airway cross-sections using a computational model for mucosal thickening based on the theory of finite growth. Our simulations indicate that smaller airways are at a higher risk of narrowing than larger airways and that regions away from a branch narrow more drastically than regions close to a branch. These results agree with clinical observations and could help explain the underlying mechanisms of progressive airway obstruction. Understanding growth-induced instabilities in constrained geometries has immediate biomedical applications beyond asthma and chronic bronchitis in the diagnostics and treatment of chronic gastritis, obstructive sleep apnea and breast cancer.

## 1. Motivation

Asthma and chronic bronchitis are the most prominent obstructive lung diseases affecting millions of people worldwide. Asthma affects 8% of the adult population and 20% of children [1]. Chronic bronchitis, a manifestation of chronic obstructive pulmonary disease, affects 25% of the adult population and is the fourth leading cause of death [2]. Asthma is generally classified as *reversible*, triggered by allergens, weather or exercise [3], while chronic bronchitis is *irreversible*, triggered by pollutants, toxins and smoke [4,5].

**Figure 1:**
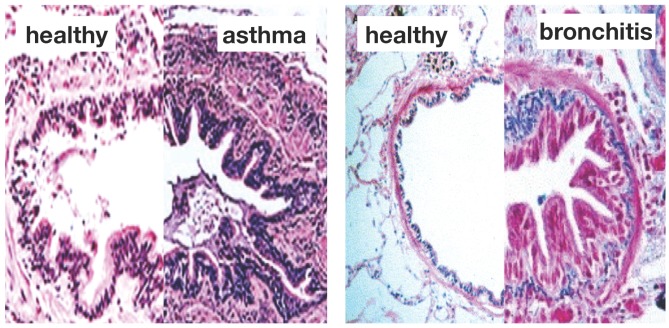
Airway wall remodeling in chronic lung disease. In contrast to the healthy airway wall, the airway wall in asthma, left, and chronic bronchitis, right, displays *airway constriction*, associated with smooth muscle thickening at the outer airway wall, and *airway inflammation*, associated with mucosal growth at the inner airway wall. These changes manifest themselves in an increase in pressure at the outer wall and an increase in volume at the inner wall, resulting in a narrowing of the lumen and progressive airflow obstruction; adapted from [1,4].

Figure 1 illustrates the two common features of asthma and chronic bronchitis: *airway constriction* associated with smooth muscle thickening at the outer airway wall; and *airway inflammation* associated with mucosal growth at the inner airway wall [6]. These two phenomena manifest themselves mechanically in an increase in pressure from active smooth muscle cell contraction and an increase in volume from an influx of mononuclear cells flooding the inner wall lining. Collectively, both phenomena initiate an inward folding of the airway wall, ultimately resulting in a narrowing of the lumen and progressive airflow obstruction [7,8].

**Figure 2:**
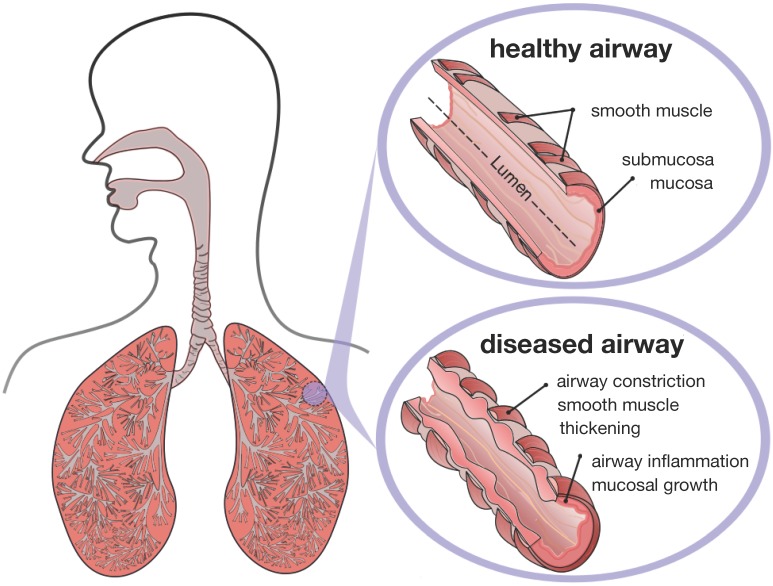
Schematic of the lungs. The trachea, the root of the respiratory tree, branches into two main bronchi, which enter the left and right lungs. In the lungs, the bronchial tree continues to branch in humans into 23 to 27 generations, yielding approximately 17 million branches. The final generation of terminal bronchioles opens into the alveolar space, where gas transfer occurs. Airway wall remodeling affects the small airways between the fourth and 14th generation. Healthy small airways are less than 2 mm in diameter, non-cartilaginous and compliant. In chronic lung disease, the small airways experience smooth muscle thickening and mucosal growth, resulting in progressive airflow obstruction.

Figure 2 displays a schematic of the human lungs. The trachea, the root of the respiratory tree, branches into two main bronchi, which enter the left and right lungs. In the lungs, the bronchial tree continues to branch in humans into 23 to 27 generations, yielding approximately 17 million branches [9]. The final generation of terminal bronchioles opens into the alveolar space, the region where gas transfer takes place [6]. Airway wall remodeling affects the small airways between the fourth and fourteenth generation [4]. Healthy small airways are less than 2 mm in diameter, non-cartilaginous and compliant [10]. In chronic lung disease, the dimensions of the small airways roughly double, resulting in a loss of structural compliance, airflow obstruction and difficulties in breathing [11].

**Figure 3:**
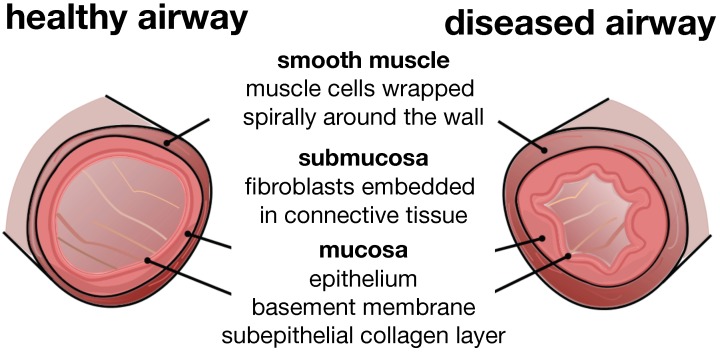
Schematic of the airway wall. Small airways consist of three distinct layers, the mucosa, the submucosa and the smooth muscle layer. In chronic lung disease, the smooth muscle layer thickens and creates an elevated pressure at the outer wall, while the mucosal layer experiences inflammation and increases in volume at the inner wall. The airway wall folds inwards, resulting in progressive airflow obstruction.

Figure 3 illustrates a typical cross-section of the small airway wall with its three distinct layers. The mucosa, the innermost layer, consists of the epithelium, the basement membrane and a subepithelial collagen layer. The stiffness of the mucosa depends primarily on the subepithelial collagen layer, whose thickness ranges from 4–5 μm in healthy to 7–23 μm in diseased airways [12]. The submucosa, the middle layer, consists of fibroblasts and proteoglycans embedded in a loose, irregular network of elastin and collagen. Although the submucosa is about an order of magnitude softer than the mucosa [6], it contributes significantly to the overall stiffness of the airway wall because of its considerable thickness. The smooth muscle layer, the outermost layer, consists of spirally arranged smooth muscle cells. In the healthy airway, these smooth muscle cells regulate the amount of airflow through active cellular contraction. In the diseased airway, smooth muscle thickening and uncontrolled muscle contraction generate an elevated contractile force, resulting in an increased wall stiffness and a decreased lumen.

The numerous fatalities and sheer number of people whose lives are impacted daily by asthma and chronic bronchitis motivate the need to understand the microstructural mechanisms of chronic lung disease. The past two decades have seen a tremendous progress in the mathematical and mechanical analysis of airway constriction and airway inflammation [6,13]. Table 1 classifies the most prominent analytical and computational models of chronic lung disease. While earlier approaches focus primarily on airway constriction and smooth muscle thickening modeled through an increased pressure of the outer layer [14,15], recent approaches focus on airway inflammation and mucosal thickening modeled through an increased volume of the inner layer [8,16]. Few studies combine both mechanisms to explore whether the associated structural alterations have a positive or negative feedback on one another [13,17].

Analytical modeling can provide valuable insight into the critical failure mode [18,19]. Using classical bifurcation analysis [20], analytical models can help identify critical conditions, such as the critical pressure [14,21] or the critical amount of growth [8,16] at the onset of failure. However, analytical models fall short of predicting the progression of failure throughout the post-failure regime [22,23]. This is particularly important, since complications in chronic lung disease are typically associated with the later stages of failure rather than with the onset of folding. In addition, analytical models are typically limited to simplified constitutive models and regular geometries. It is thus not surprising that all existing models of airway wall thickening are restricted to regular, circular cross-sections [24].

Numerical modeling cannot only predict the onset of failure, but also the structural response throughout the entire post-failure regime [25,26]. For example, they can predict the critical growth at first contact, which serves as a valuable clinical metric to characterize the severe stages of airway obstruction. Using finite element analysis, numerical models can easily incorporate more realistic constitutive models and complex patient-specific geometries [27,28]. Computational modeling has been intensely used to simulate growth within the vascular tree [29,30]. Yet, to date, there are no compelling computational models to simulate growth within the bronchial tree. This is particularly important, since the bronchial tree branches a lot more frequently than the vascular tree. In fact, airway branching occurs once every two to four airway diameters [6]. This implies that only a small fraction of the bronchial tree is in fact a circular cross-section, and irregular, non-circular cross-sections might play an important, yet underestimated, role.

**Table 1:** Classification of existing models of chronic lung disease.

analysis	microstructural mechanism	analytical solution onset of failure	numerical solution post-failure regime
symptoms			
**airway constriction**	smooth muscle thickening → increased pressure on submucosal layer	bifurcation analysis → folding pressure $p^{\mathrm{fold}}$ → number of folds $n^{\mathrm{fold}}$ [13,14,15,17]	finite element analysis → contact pressure $p^{\mathrm{crit}}$ → folded configuration [14,15]
**airway inflammation**	mucosal inflammation → volume growth of mucosal layer	bifurcation analysis → folding growth $\vartheta^{\mathrm{fold}}$ → number of folds $n^{\mathrm{fold}}$ [8,13,16,17,23,24]	finite element analysis → contact growth $\vartheta^{\mathrm{crit}}$ → folded configuration [8,16,23,25,31,32]

*We hypothesize that the geometry of the bronchial tree plays a crucial role in chronic airway obstruction and that critical failure conditions vary significantly along a branching airway segment.* To test this hypothesis, we introduce a continuum model for mucosal thickening based on the theory of finite growth [33,34] and establish a computational model for its numerical solution using nonlinear finite element analysis. We represent growth through the multiplicative decomposition of the deformation gradient [35] into an elastic tensor, which induces stress [36], and a growth tensor, which we prescribe constitutively [37,38]. To mimic chronic disease progression up to and beyond the onset of airway wall folding, we gradually increase the amount of growth [31,39]. Although the underlying concept is generally applicable to both isotropic and anisotropic growth [40,41], here, we assume that growth is purely isotropic. We represent it through a spherical tensor scaled by the amount of volume growth [42]. We embed the resulting growth model into a nonlinear finite element setting and solve it using an incremental iterative Newton–Raphson solution scheme [43,44]. In the sequel, we outline the details of our model.

The remainder of this manuscript is organized as follows: We briefly summarize the continuum and computational modeling of finite growth in Section 2. To identify critical regions of airway narrowing, we perform systematic studies along a branching airway segment in Section 3: First, we visualize the rich variety of possible failure modes along a branching airway segment by inducing prescribed failure modes in elliptical cross-sections through sinusoidal perturbations in Section 3.1. Next, we explore the role of the relative mucosal thickness by inducing natural failure modes in circular cross-sections through random perturbations in Section 3.2. Last, we explore the role of the relative mucosal thickness by inducing natural failure modes in elliptical cross-sections without perturbations in Section 3.3. We compare the results against the literature and discuss the limitations of our study in Section 4. Finally, we conclude with a brief discussion in Section 5.

## 2. Methods

### 2.1. Continuum Modeling of Growth

We denote the mapping from a point X in the healthy configuration $B_{0}$ to corresponding point x in the diseased configuration $B_{t}$ at any given time *t* by the mapping x=φ(X,t). Its material gradient $F=\nabla_{X}\varphi$ maps infinitesimal line elements dX from the healthy configuration to infinitesimal line elements dx=F·dX in the diseased configuration. Central to modeling finite growth of the airway wall is the multiplicative decomposition of the deformation gradient F into an elastic part $F{}^{e}$ and a growth part $F{}^{g}$,

(1) $$F=\nabla_{X}\varphi =F{}^{e}\cdot F{}^{g}\;.$$

The Jacobian J=det(F) maps infinitesimal volume elements dV from the healthy configuration to infinitesimal volume elements dv in the diseased configuration dv=Jdv. Similar to the deformation gradient, we decompose the total volume change *J* multiplicatively into a reversible elastic volume change $J^{e}=det\;\left(F{}^{e}\right)$ and an irreversible growth volume change $J^{g}=det\;\left(F{}^{g}\right)$,

(2) $$J=det\;\left(F\right)=J^{e}\;J^{g}\;.$$

We assume that the airway wall grows isotropically and introduce its growth tensor $F{}^{g}$ as the identity tensor I scaled by the scalar-valued growth multiplier *ϑ*,

(3) $$F{}^{g}=\vartheta \;I\;.$$

In chronic lung disease, growth is primarily driven by non-mechanical, chemical or biochemical stimuli. Here, we propose a growth rate, which exponentially evolves with time, $\dot{\vartheta }=\frac{1}{\tau }\left[\;\vartheta^{\max}-1\;\right]\;\left[\;\exp\;\left(-t/\tau \right)\;\right]$, where *t* is the time; *τ* is a time constant;and $\vartheta^{\max}$ is the asymptotic value for maximum growth at t→∞. Integrating in time yields an explicit expression for the growth multiplier,

(4) $$\vartheta =1+\left[\;\vartheta^{\max}-1\;\right]\;\left[\;1-\exp\;\left(-t/\tau \right)\;\right]\;.$$

For the particular format of the growth tensor $F{}^{g}$ in Equation (3), the growth multiplier *ϑ* takes the physical interpretation of the third root of the grown volume, $J^{g}=\vartheta^{3}$. Using the inverse of the growth tensor, $F{}^{g-1}=I/\vartheta$, we obtain explicit formulations for the elastic tensor, $F{}^{e}=F\cdot F{}^{g-1}$,

(5) $$F{}^{e}=F\;/\;\vartheta \;,$$

for the elastic Jacobian, $J^{e}=J\;/\;J^{g}$,

(6) $$J^{e}=J\;/\;\vartheta^{3}\;,$$

and for the elastic left Cauchy-Green deformation tensor, $b{}^{e}=F{}^{e}\cdot F{}^{e\;t}$,

(7) $$b{}^{e}=F\cdot F{}^{t}\;/\;\vartheta^{2}$$

as the growth-scaled total deformation gradient F, total Jacobian *J*, and total left Cauchy-Green deformation tensor $b=F\cdot F{}^{t}$. We idealize the airway wall as isotropic, hyperelastic Neo-Hookean material with Lamé constants *λ* and *μ*. Its Helmholtz free energy function,

(8) $$\psi =\frac{1}{2}\;\lambda \;\ln^{2}\left(J^{e}\right)+\frac{1}{2}\;\mu \;\left[\;I_{1}^{e}-3-2\;\ln\;\left(J^{e}\right)\;\right]\;,$$

depends exclusively on the elastic invariants $I_{1}^{e}=b{}^{e}:i$ and $J^{e}=det\left(F{}^{e}\right)$, where i is the spatial unit tensor. The Kirchhoff stress,

(9) $$\tau =2\;\frac{\partial \psi }{\partial b{}^{e}}\cdot b{}^{e}=\left[\;\lambda \;\ln\;\left(J^{e}\right)-\mu \;\right]\;i+\mu \;b{}^{e}\;,$$

follows thermodynamic considerations, and introduces the Eulerian constitutive moduli,

(10) $$c=4\;b{}^{e}\cdot \frac{\partial^{2}\psi }{\partial b{}^{e}⊗\partial b{}^{e}}\cdot b{}^{e}=\lambda \;i⊗i+\left[\;\mu -\lambda \;\ln\left(J^{e}\right)\right]\left[\;i\;\bar{⊗}\;i+i\;\underline{⊗}\;i\right]\;.$$

Here we have used the following abbreviations for the non-standard fourth order tensor products according to ${\left\{•⊗\circ \right\}}_{ijkl}={\left\{•\right\}}_{ij}\;{\left\{\circ \right\}}_{kl}$ and ${\left\{•\bar{⊗}\circ \right\}}_{ijkl}={\left\{•\right\}}_{ik}\;{\left\{\circ \right\}}_{jl}$ and ${\left\{•\underline{⊗}\circ \right\}}_{ijkl}={\left\{•\right\}}_{il}\;{\left\{\circ \right\}}_{jk}$.

### 2.2. Computational Modeling of Growth

To solve the underlying boundary value problem, we implement the equations of finite growth as a constitutive subroutine into a nonlinear finite element solver. For every time step, we successively calculate the growth multiplier *ϑ* from Equation (4), the growth tensor $F{}^{g}$ from Equation (3), the elastic tensor $F{}^{e}$ from Equation (5), the elastic left Cauchy-Green tensor $b{}^{e}$ from Equation (7), the Kirchhoff stress τ from Equation (9), and the modified Eulerian tangent moduli c from Equation (10). The constitutive subroutine returns the local stress τ and the tangent moduli c, which enter the global righthand side vector and iteration matrix for the global Newton-Raphson iteration.

Since the common underlying mechanism of mucosal folding is a nonlinear instability problem, the computational solution is highly sensitive, in particular at the onset of failure. It proves critical to adopt an adaptive time stepping scheme, which decreases the time step size in the proximity of the instability. Here we automatically adjust the time step size in response to the convergence behavior of the global Newton Raphson iteration. In particular, we divide the current time step size by two if more than six Newton iterations are required to reach the incremental equilibrium state within a tolerance of $10^{-10}$, and otherwise increase the current time step size by one fourth.

## 3. Results

**Figure 4:**
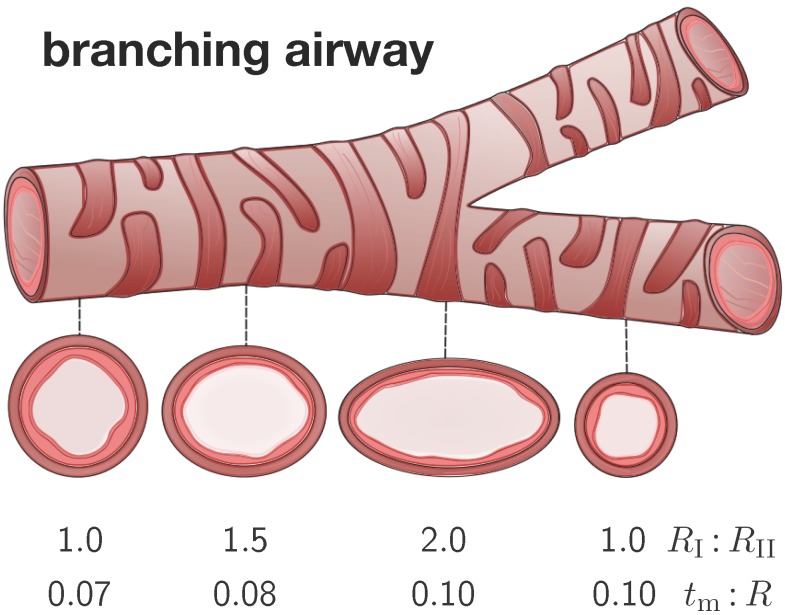
Branching airway segment. Along the bronchial tree, the airway cross varies substantially in geometry. We characterize each cross-section through the semi-major-to-semi-minor-axis ratio $R_{I}:R_{\mathrm{II}}$ and through the mucosal-thickness-to-radius ratio $t_{m}:R$. The ratio $R_{I}:R_{\mathrm{II}}$ is larger close to and smaller away from a branching region. The ratio $t_{m}:R$ is smaller close to and larger away from the trachea.

To identify critical regions of airway narrowing, we perform systematic parameter studies for varying cross-sections within the bronchial tree. Figure 4 illustrates four representative elliptical cross-sections along a branching airway segment. In analogy with the literature [8,15], we introduce the airway radius *R* as the distance from the airway center to the closest point on the mucosal-submucosal interface. We denote the mucosal and submucosal thicknesses as $t_{m}$ and $t_{s}$, such that the inner and outer radius of a circular cross-section are $R-t_{m}$ and $R+t_{s}$. For elliptical cross-sections, we characterize the degree of ellipticity through the semi-major and semi-minor axes $R_{I}$ and $R_{\mathrm{II}}$ of the submucosal-muscular interface, where the semi-minor axis is identical to the outer radius of submucosal layer $R_{\mathrm{II}}=R+t_{s}$. We model the mucosal and the submucosal layers as elastically incompressible with stiffnesses $\mu_{m}$ and $\mu_{s}$ and the idealize the smooth muscle layer as rigid. While the mucosa is allowed to grow at a speed of *τ* towards a maximum growth of $\vartheta^{\max}$, the submucosa remains purely elastic.

To characterize the individual cross-sections in terms of non-dimensional parameters, we introduce the mucosal-thickness-to-radius ratio $t_{m}:R$, the submucosal-thickness-to-radius ratio $t_{s}:R$, the ellipticity ratio $R_{I}:R_{\mathrm{II}}$, and the mucosal-to-submucosal stiffness ratio $\mu_{m}:\mu_{s}$. Since previous studies have shown that the failure mode is more sensitive to the ratio $t_{m}:R$ than to the ratios $t_{s}:R$ and $\mu_{m}:\mu_{s}$ [8,15], we fix the latter two to $t_{s}:R=0.5$ and $\mu_{m}:\mu_{s}=10:1$ [6]. In the following studies, we vary the relative mucosal thickness ratio between $t_{m}:R=\left[\;0.01,\ldots ,0.20\;\right]$ and the ellipticity ratio between $R_{I}:R_{\mathrm{II}}=\left[\;1.00,\ldots ,2.25\;\right]$.

We discretize each cross-section using classical tri-linear brick elements and assume plane strain conditions along the airway’s long axis. Since the smooth muscle layer is significantly stiffer than the mucosal and submucosal layers, we represent it implicitly through homogeneous Dirichlet boundary conditions at the submucosal-mucosal interface. We allow the mucosal layer to grow and fold inward. Once we detect the first contact between two folds, we terminate the simulation and record the corresponding growth factor as the critical growth at first contact. To explore regional variations of growth along the bronchial tree, we perform three sets of simulations: In Section 3.1, for varying elliptical cross-sections with *sinusoidal perturbations*, we demonstrate how growth drives the solution along the prescribed failure mode, which is *a priori* defined by the corresponding perturbation. In Section 3.2, for circular cross-sections and *random perturbations*, we demonstrate how growth drives the solution into the natural failure mode, which is governed by the relative mucosal thickness. In Section 3.3, for varying elliptical cross-sections *without perturbations*, we demonstrate how growth drives the solution into the natural failure mode, which is induced naturally by curvature heterogeneity and governed by the relative mucosal thickness.

### 3.1. Sensitivity of Failure Mode with Respect to Ellipticity

**Figure 5:**
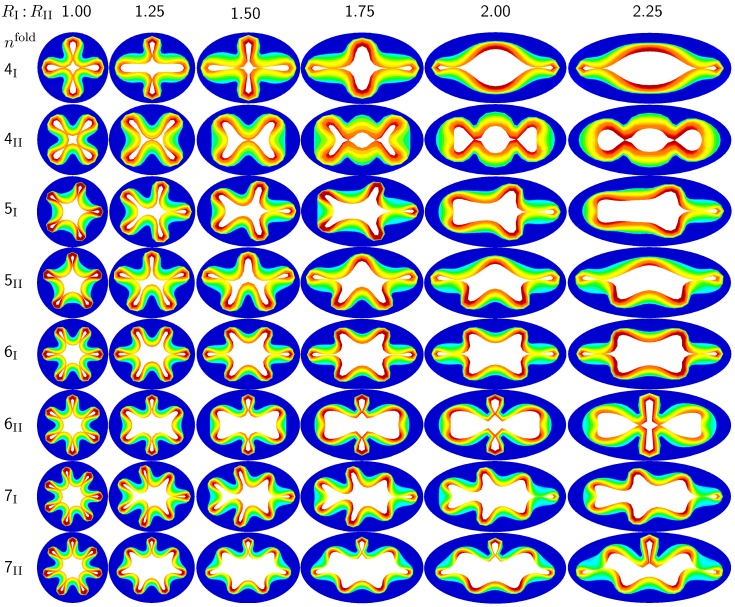
Sensitivity of the failure mode with respect to ellipticity. Smaller ellipticity ratios represent circular cross-sections away from a branching region; larger ellipticity ratios represent elliptical cross-sections close to a branching region. With increasing ellipticity, the heterogeneity of the failure mode increases. With increasing heterogeneity, the lumen area at the first contact remains significantly larger. This might indicate that circular cross-sections away from the branching region are at a higher risk of to airflow obstruction than elliptical cross-sections close to a branching region.

The first set of examples in Figure 5 visualizes the wealth of failure modes for different ellipticity ratios. From a mechanical point of view, this study is closely related to the buckling of elliptical tubes under radial compression [45]. We explore six different cross-sections with ellipticity ratios varying as $R_{I}:R_{\mathrm{II}}$=[1.00,1.25,1.50,1.75,2.00,2.25], where the limits of $R_{I}:R_{\mathrm{II}}=1.00$ and $R_{I}:R_{\mathrm{II}}=2.25$ mimic cross-sections away from and close to a branching region. To trigger a specific, *prescribed failure mode*, we perturb the homogeneous elliptical cross-section with *sinusoidal perturbations* of a magnitude of one fourth of the mucosal thickness, 0.25 $t_{m}$, and a frequency of $n^{\mathrm{fold}}$ modes along the inner mucosal surface. To visualize potential failure modes, we vary the mode number as $n^{\mathrm{fold}}=\left[\;4,5,6,7\;\right]$ and perform two simulations for each mode: Simulation I is at least symmetric to the semi-major axis and has at least one fold along the axis of symmetry; Simulation II is at least symmetric to the semi-minor axis.

Figure 5 illustrates the different failure modes for varying ellipticity ratios $R_{I}:R_{\mathrm{II}}$ and varying mode numbers $n^{\mathrm{fold}}$. The color code reflects the circumferential stress with red values at the inner mucosa corresponding to high circumferential traction and blue values at the outer submucosa corresponding to low traction. As the ellipticity ratio increases, from left to right, the heterogeneity of the failure mode increases. Notably, all folding patterns, from top to bottom, display a discontinuous alteration in the underlying failure mode as the ellipticity increases: Mode $4_{I}$, displayed in the first row, changes from a double horizontal double vertical contact mode at $R_{I}:R_{\mathrm{II}}=1.00$ via a double horizontal contact mode at $R_{I}:R_{\mathrm{II}}=1.25$ to a double vertical contact mode at $R_{I}:R_{\mathrm{II}}\geq 1.50$. Mode $4_{\mathrm{II}}$, displayed in the second row, changes from a quadruple-diagonal contact mode at $R_{I}:R_{\mathrm{II}}=1.00$ via a single vertical contact mode at $R_{I}:R_{\mathrm{II}}=1.25$ and $R_{I}:R_{\mathrm{II}}=1.50$ to a double vertical contact mode at $R_{I}:R_{\mathrm{II}}\geq 1.75$. Mode $6_{\mathrm{II}}$, displayed in the sixth row, changes from a sextuple contact mode at $R_{I}:R_{\mathrm{II}}=1.00$ via a double horizontal contact mode from $R_{I}:R_{\mathrm{II}}=1.25$ to $R_{I}:R_{\mathrm{II}}=2.00$ to a double vertical contact mode at $R_{I}:R_{\mathrm{II}}=2.25$. Mode $7_{\mathrm{II}}$, displayed in the eighth row, changes from a septuple contact mode at $R_{I}:R_{\mathrm{II}}=1.00$ via a single vertical contact mode from $R_{I}:R_{\mathrm{II}}=1.25$ to $R_{I}:R_{\mathrm{II}}=2.00$ to a double horizontal contact mode at $R_{I}:R_{\mathrm{II}}=2.25$. With increasing ellipticity, from left to right, the lumen area at the first contact remains significantly larger. This might indicate that circular cross-sections away from the branching region are at a higher risk of airflow obstruction than elliptical cross-sections close to a branching region.

### 3.2. Sensitivity of the Failure Mode with Respect to Relative Mucosal Thickness

**Figure 6:**
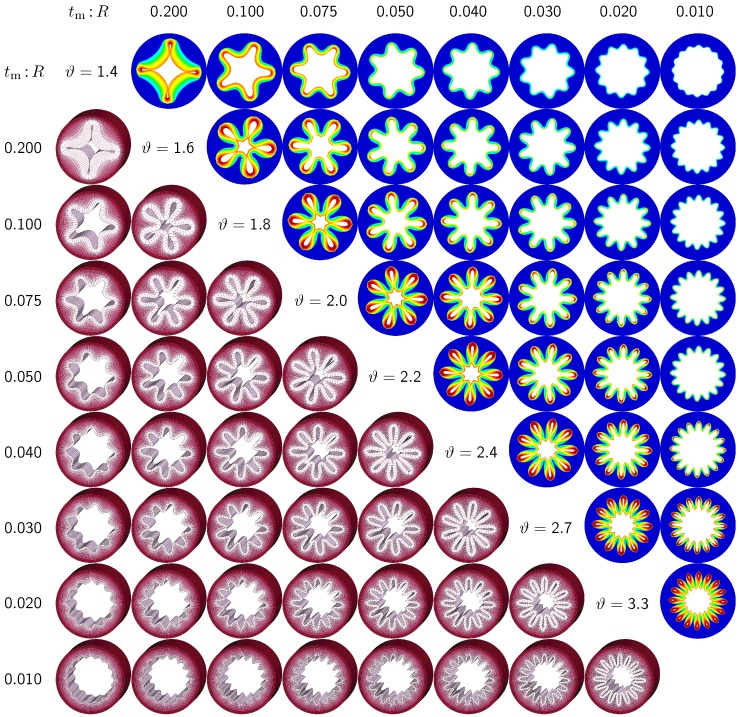
Sensitivity of failure mode with respect to relative mucosal thickness for a circular cross-section with $R_{I}:R_{\mathrm{II}}=1.0$. Larger relative mucosal thicknesses represents smaller bronchi away from the trachea; smaller relative mucosal thicknesses represents larger bronchi close to the trachea. With increasing relative mucosal thickness, the number of folds decreases. With the decreasing number of folds, the critical growth *ϑ* at the first contact becomes significantly smaller. This might indicate that smaller bronchi with a larger relative mucosal thickness are at a higher risk of airflow obstruction than larger bronchi with a smaller relative mucosal thickness.

The second set of examples in Figure 6 probes the sensitivity of the failure mode for different relative mucosal thickness ratios in a circular cross-section with $R_{I}:R_{\mathrm{II}}=1.0$. From a mechanical point of view, this study is closely related to the buckling analysis of circular tubes under an external pressure [17]. We explore eight different cross-sections with relative mucosal thickness ratios varying as $t_{m}:R=\left[\;0.200,0.100,0.075,0.050,0.040,0.030,0.020,0.010\;\right]$. As illustrated in Figure 4, variations of $t_{m}:R$ occur not only in similar generations of bronchi with the same radius *R* and varying initial mucosal thickness $t_{m}$, but also in different airway generations along the bronchial tree with varying radius *R* and similar mucosal thickness $t_{m}$. In fact, the overall wall-thickness-to-radius ratio was measured to vary from 0.044 in the first generation of bronchi to 0.075 in the sixteenth generation [6], indicating that the relative thickness increases as we descend down the bronchial tree. To trigger the *natural failure mode*, we perturb the homogeneous circular cross-section with *random perturbations* of one percent of the mucosal thickness, 0.01 $t_{m}$. To mimic the onset of failure, we take snap shots at ϑ=[1.4,1.6,1.8,2.0,2.2,2.4,2.7,3.0], which correspond to the first points of contact of the eight different cross-sections.

Figure 6 illustrates the evolution of the different failure modes for varying relative mucosal thicknesses $t_{m}:R$ and for different first points of contact *ϑ*. The upper right section displays the circumferential stress; the lower left section displays the three-dimensional failure mode, both synchronized at the same time point. Figure 6 demonstrates that the relative mucosal thickness determines the failure mode and the number of folds, irrespective of the small initial random perturbation. As growth progresses, the folds become more and more pronounced until first contact occurs.

Figure 6 confirms that circular cross-sections with random perturbations can generate both even and odd failure modes. As the relative mucosal thickness decreases, the number of folds increases from $n^{\mathrm{fold}}=4$ at $t_{m}:R=0.200$ to $n^{\mathrm{fold}}=16$ at $t_{m}:R=0.010$. For cross-sections with relatively thin mucosal layers, towards the right column and the bottom row, more growth is required to form the first contact. The overall mucosal thickness at this first contact point, however, is remarkably similar for all eight cross-sections, as shown along the diagonal. In summary, with increasing relative mucosal thickness, from top right to left, the required growth to form the first contact decreases. This might indicate that smaller bronchi with larger relative mucosal thickness, displayed towards the left, are at a higher risk of airflow obstruction than larger bronchi with a smaller relative mucosal thickness, displayed towards the right.

### 3.3. Sensitivity of Failure Mode with Respect to Ellipticity and Relative Mucosal Thickness

**Figure 7:**
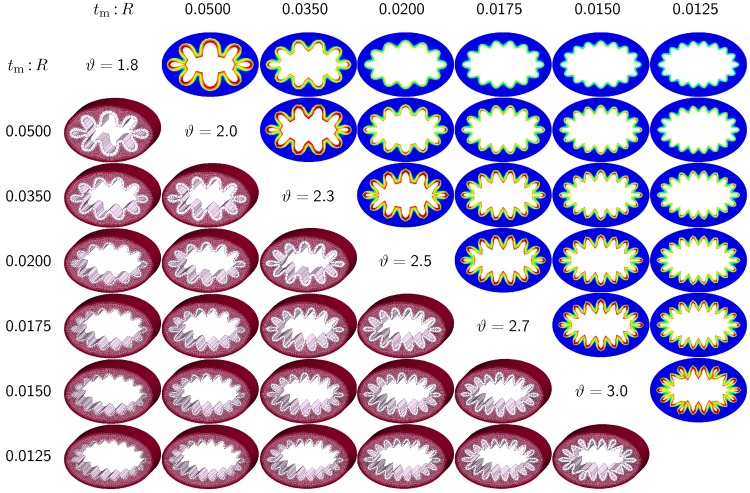
Sensitivity of the failure mode with respect to relative mucosal thickness for moderately elliptical cross-section with $R_{I}:R_{\mathrm{II}}=1.5$. The instability occurs naturally at regions of highest curvature along the semi-major axis and propagates inward until it reaches the semi-minor axis. First contact occurs at regions of highest curvature and induces a moderately localized failure mode. This might indicate that airway obstruction is less drastic in moderately elliptical cross-sections than in circular cross-sections.

**Figure 8:**
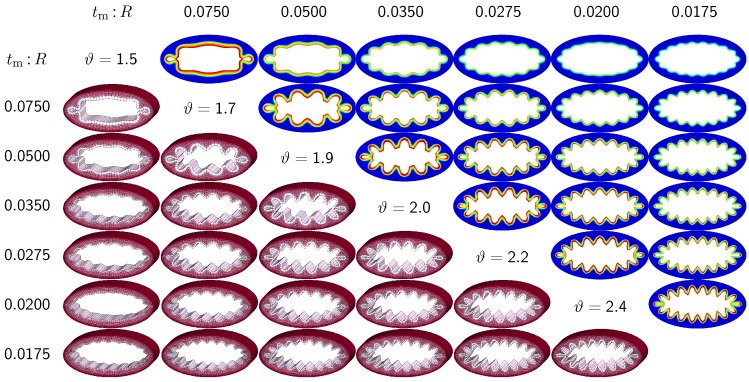
Sensitivity of the failure mode with respect to relative mucosal thickness for severely elliptical cross-section with $R_{I}:R_{\mathrm{II}}=2.0$. The instability occurs naturally at regions of highest curvature along the semi-major axis and propagates inward until it reaches the semi-minor axis. First contact occurs at regions of highest curvature and induces a severely localized failure mode. This might indicate that airway obstruction is less drastic in severely elliptical cross-sections than in circular and moderately elliptical cross-sections.

The third set of examples in Figure 7 and Figure 8 probes the sensitivity of the failure mode for both different ellipticity ratios and different relative mucosal thickness ratios. We compare a moderately elliptical cross-section with $R_{I}:R_{\mathrm{II}}=1.50$ and a severely elliptical cross-section with $R_{I}:R_{\mathrm{II}}=2.00$ to the circular cross-section with $R_{I}:R_{\mathrm{II}}=1.00$ discussed in the previous subsection. For each cross-section, we vary the relative mucosal thickness as $t_{m}:R=\left[\;0.075,0.050,0.035,0.0275,0.020,0.0175,0.015,0.0125\;\right]$. Elliptical cross-sections display a natural heterogeneity as the curvature varies along the circumference. To trigger the *natural failure mode*, we can thus simulate the plain elliptical cross-section *without perturbations*. To mimic the onset of failure, we take snap shots at ϑ=[1.8,2.0,2.3,2.5,2.7,3.0] for the moderately elliptical cross-section and at ϑ=[1.5,1.7,1.9,2.0,2.2,2.4] for the severely elliptical cross-section. These correspond to the first points of contact of the six different cross-sections of each set.

Figure 7 and Figure 8 illustrate the evolution of the different failure modes for varying relative mucosal thickness ratios $t_{m}:R$ and for different first points of contact *ϑ*. Similar to Figure 6, the upper right section displays the circumferential stress; the lower left section displays the three-dimensions of the failure mode. Again, red circumferential stress values at the inner mucosa correspond to high traction and blue values at the outer submucosa correspond to low traction. In elliptical cross-sections, the curvature is highest towards the long ends of the semi-major axis. This is where the initial instability occurs naturally. As growth progresses, the instability gradually propagates inward along the semi-major axis and forms additional folds until it reaches the semi-minor axis. As growth progresses further, the folds become more and more pronounced until first contact occurs. For regular ellipses, first contact always occurs at the long ends of the semi-major axis. Because of their inherent geometric heterogeneity, elliptical cross-sections tend to fold naturally in a double-symmetric folding pattern with naturally occurring outward folds along their semi-major axis. This implies that unperturbed simulations of elliptical cross-sections only generate even failure modes, $n^{\mathrm{fold}}=\left[\;8,10,12,14,16,18\;\right]$. As the relative mucosal thickness decreases, the number of folds increases from $n^{\mathrm{fold}}=8$ at $t_{m}:R=0.050$ and $t_{m}:R=0.075$ to $n^{\mathrm{fold}}=18$ at $t_{m}:R=0.0125$ and $t_{m}:R=0.0175$, illustrated in the top and bottom rows of Figure 7 and Figure 8. In agreement with Section 3.2, cross-sections with a relatively thin mucosal layer, towards the right column and the bottom row, require more growth to form the first contact. As the ellipticity increases, $R_{I}:R_{\mathrm{II}}=\left[\;1.0,1,5,2.0\right]$ the underlying failure mode at the first contact becomes more localized. Accordingly, cross-sections with larger ellipticity, from Figure 6 through Figure 8, require less growth to form the first contact. However, in agreement with Section 3.1, since contact occurs only locally in cross-sections with larger ellipticity, the lumen area at the first contact remains significantly larger. This might indicate that circular cross-sections obstruct more drastically than elliptical cross-sections.

**Figure 9:**
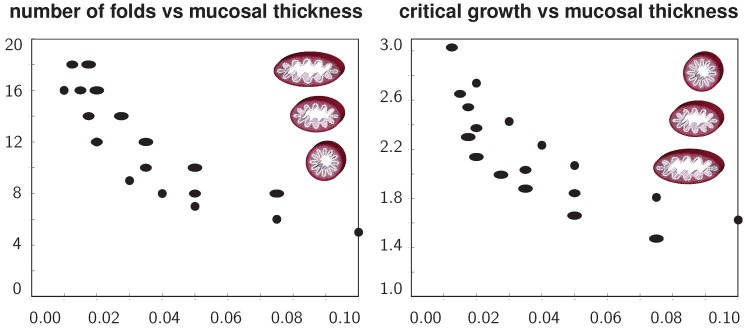
Number of folds and critical growth for varying ellipticities and varying relative mucosal thicknesses. With increasing relative mucosal thickness, the number of folds and the critical growth at the first contact decrease. With increasing ellipticity, the number of folds increases, while the critical growth decreases. This might indicate that smaller airways are at a higher risk of airflow obstruction than larger airways and that circular cross-sections obstruct more drastically than elliptical cross-sections.

Figure 9 summarizes the above observations in two graphs to quantify the number of folds and the critical growth for varying ellipticity and varying relative mucosal thickness. With increasing relative mucosal thickness, the number of folds and the critical growth at the first contact decrease. With increasing ellipticity, the number of folds increases, while the critical growth at the first contact decreases. In view of the folded elliptical configurations displayed in Figure 5, Figure 7 and Figure 8, this decrease of critical growth seems less alarming, since contact occurs only locally at the two outermost folds, while the lumen area remains relatively large. Overall, the two graphs confirm that smaller airways are at a higher risk of airflow obstruction than larger airways and that circular cross-sections obstruct more drastically than elliptical cross-sections.

## 4. Discussion

The objective of this study was to elucidate the role of mechanics in chronic lung disease. We have reviewed the key mechanisms of asthma and chronic bronchitis: airway constriction associated with smooth muscle thickening and airway inflammation associated with mucosal growth. Since the recent literature focuses primarily on the latter mechanism, we have restricted our analysis exclusively to mucosal growth. We have summarized the continuum modeling of finite growth and its computational realization using finite element analysis. Our computational model allowed us to explore the pre- and post-failure behavior of cross-sections of varying ellipticities and varying relative mucosal thicknesses to identify critical regions of airway narrowing along the bronchial tree. Our simulations confirmed our hypothesis that the geometry of the bronchial tree plays a crucial role in chronic airway obstruction and that critical failure conditions vary significantly along a branching airway segment. In particular, we found that smaller bronchi away from the trachea are at a higher risk of airflow obstruction than larger bronchi close to the trachea and that circular cross-sections away from a branching region obstruct more drastically than elliptical cross-sections close to a branching region.

### 4.1. Comparison with Previous Studies

The sensitivity of the failure mode with respect to the relative mucosal thickness in circular cross-sections has been studied intensely throughout the past two decades. Studies of airway constriction, represented through an elevated external pressure, revealed similar trends as the present study [14,15]. In particular, these analytical studies demonstrated an exponential decay of the number of folds with an increasing relative mucosal thickness [6], in agreement with Figure 9. Our computationally predicted failure mode of $n^{\mathrm{fold}}=5$ at $t_{m}:R=0.1$, illustrated in Figure 6, agrees nicely with the analytically predicted value [15]. However, our computational solution behaves slightly more stiffly than the analytical solution, and the number of folds increases slower with decreasing mucosal thicknesses than predicted analytically. This discrepancy might be attributed to both the relatively coarse finite element discretization and the use of lower order elements, which collectively behave poorly in the context of bending.

Studies of airway inflammation, represented through mucosal growth, also agree nicely with the present study, both analytically [8,17] and numerically [8,16]. Again, the exponential decay of the number of folds for an increasing relative mucosal thickness is in nice agreement with Figure 9. Yet, the decay is slightly more pronounced in the literature, most likely because of the use of enhanced hybrid elements [8]. The problem of airway folding falls into the broad category of constrained growth in tubular geometries, which has recently received increasing attention [25]. For example, recent studies of folding of intestinal villi have revealed a similar exponential dependence of the number of folds on the thickness of the growing layer [22]. Overall, there is general agreement that the relative mucosal thickness is a critical parameter in chronic lung disease [6]: bronchial cross-sections with a larger relative mucosal thickness typically generate less folds [46] and require a smaller amount of growth to narrow than cross-sections with a smaller relative mucosal thickness. These findings are in general agreement with the clinical observation that smaller airways with a larger relative mucosal thickness are more severely affected by airway obstruction than larger airways [10,12,46]. This agrees nicely with Figure 6 and Figure 9 and with the results documented in Section 3.2 and Section 3.3 of the current study.

While many studies focus on the sensitivity of the failure mode for varying mucosal thicknesses in circular cross-sections, the sensitivity of the failure mode for varying ellipticities has long been underestimated and remains severely understudied [6]. This is not surprising, since most existing models are based on analytical or semi-analytical solutions, which are typically restricted to idealized circular or tubular geometries [24]. However, cross-sectional ellipticity seems to play a critical role in airway obstruction. Since the bronchial tree branches once every two to four airway diameters, only a small fraction of bronchi is of a circular cross-section [6]. In pulmonary fluid mechanics, airway branching has long been recognized to play an important role [47]. Yet, in pulmonary solid mechanics, it has been conveniently neglected to date. Because of their lower symmetry, elliptical cross-sections display a richer variety of failure modes, as illustrated in Figure 5. By triggering specific failure models through small sinusoidal perturbations, we were able to create both even and odd elliptical failure modes through constrained growth. In particular, failure modes $4_{I}$, $5_{I}$, $5_{\mathrm{II}}$ and $6_{\mathrm{II}}$, illustrated in the first, third, fourth and fifth rows in Figure 5, agree nicely with the most relevant shell-type failure modes, 9, 11, 10 and 13, reported for elliptical hollow sections under external pressure [45]. Overall, we conclude that cross-sectional ellipticity is another critical parameter in chronic lung disease: bronchial cross-sections with a larger ellipticity require a smaller amount of growth to form the first contact than cross-sections with a smaller ellipticity. However, the failure mode becomes more localized with increasing ellipticity, and the cross-section narrows less drastically. These general observation are documented in Figure 6 to Figure 9 and throughout Section 3.1 and Section 3.3 of the current study.

### 4.2. Limitations

In the clinical literature, chronic alterations in the diseased airway wall are collectively referred to as airway wall remodeling. Our computational predictions of mucosal folding, progressive lumen narrowing and airflow obstruction are in general agreement with clinical observations during airway wall remodeling in both asthma [1] and chronic bronchitis [4]. For conceptual simplicity, we have initiated these alterations exclusively through the growth of the mucosal layer, while in reality, airway wall remodeling is a multifactorial process. In future studies, we will include the effects of the thickening of the smooth muscle layer [13,32] and of gradual alterations in composition across all three layers [3]. A more realistic model might also require us to incorporate the effects of anisotropic elasticity and anisotropic growth [41,48], for example, through an independent representation of surface growth [43,49] and thickness growth [40,44]. Since pathological studies have reported growth to be localized primarily in the basement membrane [3], we could even represent the basement membrane as a growing surface equipped with its own potential energy [31,39]. Ultimately, it would be interesting to show how mucosal growth is correlated to prestrain and residual stress in the airway wall [50], concepts that have recently been identified to play a crucial role in living systems in health and disease [51].

While our model is inherently three-dimensional, the results discussed here assume plane strain conditions. This implies that growth-induced instabilities are restricted to cross-sectional folding and do not account for longitudinal folding modes [22,23]. However, the simulation of general three-dimensional failure modes is conceptually straightforward, although, of course, computationally more expensive. From a numerical point of view, the next step would be to adopt softer, enhanced elements to represent incompressibility [52] and bending [8] more accurately. This will become crucial when moving to three-dimensional simulations of physiologically realistic airway bifurcations [47] and, eventually, of image-based patient-specific models. While all these refinements are reasonable and logical next steps, we believe that they will not affect the general trends of the present study.

## 5. Conclusions

Chronic lung disease is the fourth leading cause of death, and mild airflow obstruction affects more than one quarter of the adult population. Mechanical factors induced through smooth muscle thickening and mucosal growth play a crucial role in chronic lung disease. Yet, the spatio-temporal evolution of mechanical factors along the bronchial tree is not fully understood. Here, we have adopted a computational model for finite growth to identify critical regions of airway narrowing along a branching airway segment. Our simulations indicate that smaller bronchi are at a higher risk of airflow obstruction than larger bronchi and that circular cross-sections obstruct more drastically than elliptical cross-sections. These results agree excellently with clinical observations and could help explain the underlying mechanisms of progressive airway narrowing. Understanding growth-induced instabilities in constrained geometries could have immediate biomedical applications beyond asthma and chronic bronchitis in the diagnostics and treatment of gastritis, obstructive sleep apnea and breast cancer.
